# From shared decision making to patient-centered decision making

**DOI:** 10.1186/2045-4015-1-6

**Published:** 2012-01-30

**Authors:** Harvey V Fineberg

**Affiliations:** 1 Institute of Medicine, 500 Fifth Street, NW, Washington, DC 20001, USA

## Abstract

Shared decision making involving patients and physicians has gained adherents in Israel and other countries and has many virtues. This commentary argues that medical decision making should ideally be shaped by the particular needs and preferences of the patient, which may be to share in decision making, or at times call for a physician to assume full responsibility for decisions or, at the other extreme, to support and guide a patient who wishes to decide autonomously on what to do.

This is a commentary on http://www.ijhpr.org/content/1/1/5/

## Commentary

In an illuminating paper on the status of shared decision making in Israel, Talya Miron-Shatz and her colleagues review the legal protections of patient rights and dignity in clinical encounters, the requirements for lay participation in the formulation of certain health policies, and the state of education and research on the principles and practice of shared decision-making in Israel [1]. The article covers some special circumstances, such as end of life care, and examines the barriers to more widespread adoption of shared decision making, including attitudes of ambivalence on the part of physicians and patients.

The authors favor shared decision making because greater patient involvement represents higher ethical standards and because of practical advantages of improved adherence to treatment, greater satisfaction on the part of patients, and, occasionally, superior health outcomes. The paper points out that shared decision making has gained in popularity in many countries and argues that the education of health professionals should be geared toward preparing them to practice in ways that directly involve patients in their care decisions. The legislative and research infrastructure to facilitate shared decision making are currently in place in Israel, the authors argue, and Israel's universal coverage and relatively small number of health plans establish a foundation for more widespread reliance on shared decision making.

The concept of shared decision making impinges on many aspects of health care and policy, from informed consent for surgical procedures to public hearings on policy proposals. In this brief commentary, I offer a perspective on shared decision making in the clinical relationship between doctors and patients. I believe that an ideal approach to shared decision making would be more flexible than the authors of the paper present and, where appropriate, more systematic and explicit in combining the knowledge of physicians with the preferences and values of patients.

Medical encounters typically involve two parties: a patient and a doctor. One decision after another ensues. What questions to ask? What tests to perform? What advice to give? What medications to prescribe? What surgery to perform? The questions and decisions go on and on, in an endless succession of choices and actions. One way of thinking about the doctor-patient encounter is to frame it in just this way, as a set of decisions made by the doctor, the patient, or the two together.

The doctor-patient relationship is inherently asymmetrical, unlike the relationship between two business partners, or siblings, or spouses. In all of these cases, each party has information the other lacks, but in the business or family relationships, the information is often symmetrical--the two parties' respective knowledge of the situation, interests and preferences. In the case of the doctor and patient, the type of information each has is fundamentally different from that possessed by the other. The doctor has expertise, medical knowledge, and a capacity to assess the nature of disease, its severity and prognosis. The patient has personal preferences about willingness to endure pain, willingness to risk death or disability to achieve a cure, and attitudes more generally toward risk. Medical encounters may also have an overlay of emotion and distress, and involve family members in ways that complicate clear thinking and good judgment.

Studies, such as those cited by the authors, often criticize paternalistic (or authoritarian) styles of medical decision making because many patients say they favor being directly involved in their care decisions. In a survey of hospitalized and ambulatory patients in Israel, for example, the authors found that 60% of Israeli patients preferred to be involved with their physicians in making decisions, 20% preferred to make their own decisions, and an equal fraction wanted their physician to make the decisions.

Rather than insist that physicians engage patients in decisions, as preferred by a majority in such surveys, a more patient-centered strategy would prepare physicians to sensitively explore with patients their understanding and desires, and then adopt whatever way and degree of patient involvement each patient prefers. Just as it would be sub-optimal to impose a paternalistic style on the 60% of patients who prefer to be involved in their care decisions, it is equally sub-optimal to force a shared role on the 40% who prefer either to make autonomous decisions or to have their doctor decide what would be best. The key to effective training is not to educate physicians to adopt a particular style of practice (authoritarian, inclusive, or supportive). Rather, the goal should be to prepare physicians to identify and assess each patient's preferences and to adopt the style that meets the needs of each individual patient.

Shared decision making can be done well or poorly, just as surgery or any other clinical intervention. In a superior process of shared decision making, both the patient and the physician would be prepared to do their part. Health literacy among patients is a function not simply of the clarity of a doctor's communication, but also depends on one's ability to understand risk and to absorb and interpret information, part of one's general education. Here again, physicians should be prepared to adapt their communications to the capacities and backgrounds of individual patients, without being presumptuous or condescending. Physician training should likewise cover both formal and informal aspects of decision making, including methods to structure a difficult clinical choice, to select and apply relevant health literature, and to combine probabilities derived from data and expert assessment with the utilities or preferences that can only come from patients [2]. The aim, implicitly or explicitly, is to take full account of clinical knowledge and expertise jointly integrated with the attitudes toward risk and valuation of different possible outcomes that can only come from patients.

It is neither an ethical lapse nor a character flaw for a patient to prefer to have the doctor decide what to do. Nor is it irrational for a patient to want to be in control of decisions, with the physician or physicians acting as advisors and guides. Indeed, the same patient at different stages of life or facing different circumstances of illness may prefer at times to be a passive recipient of care, an active partner in decision-making, or fully in control of the choices to be made. A caring and sensitive clinician will not impose his or her own preferred style--whether authoritarian or inclusive--on the patient. Rather, the capable and humane clinician will adapt a role and mode of interaction that suits the needs of each patient at each particular time. This is patient-centered decision making. It requires a readiness on the part of physicians to involve the patient in shared decision making without feeling uniformly compelled to do so.

An ideal model of shared decision making in clinical care, in Israel and in other countries, would be patient-centered and systematic. In this mode of practice, physicians would adapt their style of practice to the needs of each particular patient. Physicians would be prepared to use both informal and formal methods of decision making, with the aim of doing what is best in each case, informed by the combination of patient preferences and the state of medical knowledge. If Israel can lead the way toward more universal patient-centered care and decision making, then it will have set a high standard for patients and doctors everywhere.

## Competing interests

The author declares that he has no competing interests.

## Author's information

HVF is president of the Institute of Medicine. He formerly served as provost of Harvard University and as dean of the Harvard School of Public Health.
